# Moving from knowledge to practice: is it time to move from teaching evidence-based medicine (EBM) to knowledge translation competency?

**DOI:** 10.1007/s40037-013-0050-7

**Published:** 2013-03-30

**Authors:** Imad S. A. Hassan

**Affiliations:** Department of Medicine 1443, King Abdulaziz Medical City, P.O. Box 22490, Riyadh, 11426 Kingdom of Saudi Arabia

Dear Sir,

Since its arrival to the scene of medicine and despite its worldwide acceptance as a new paradigm in healthcare, the implementation of evidence has been extremely slow [1, 2]. The latter was the primary driving force for the birth of the science of knowledge translation—the practical implementation of evidence [3]. The article by Widyahening et al. [4] is a welcome move for incorporating evidence-based medicine (EBM) in medical school curricula in developing countries. However, the emphasis should not be on teaching the science of EBM but rather on its practical application to patient care. As an educator of EBM, I strongly believe in including sessions on methods for redesigning daily routines to seamlessly incorporate EBM in the decision process, for example in morning meetings, ward rounds, outpatients, mortality case reviews etc. I also believe in including practical sessions on process change skills and in knowledge translation tools such as the use of integrated care pathways, order sets, other decision support tools, system redesign, etc. as part and parcel of EBM curricula. Emphasis on literature searching, appraisal, etc. without education and training on effective evidence-implementation tools may not be conducive to the recognition of the full objectives of the EBM science. On the whole, the curriculum should better be named Knowledge Translation Curriculum.
